# Integrative Analysis of Multi-Omics and Genetic Approaches—A New Level in Atherosclerotic Cardiovascular Risk Prediction

**DOI:** 10.3390/biom11111597

**Published:** 2021-10-28

**Authors:** EIena I. Usova, Asiiat S. Alieva, Alexey N. Yakovlev, Madina S. Alieva, Alexey A. Prokhorikhin, Alexandra O. Konradi, Evgeny V. Shlyakhto, Paolo Magni, Alberico L. Catapano, Andrea Baragetti

**Affiliations:** 1 Almazov National Medical Research Centre, 197341 Saint Petersburg, Russia; el.lenkin@yandex.ru (E.I.U.); alex.yakovlev@mail.ru (A.N.Y.); alieva_ms@almazovcentre.ru (M.S.A.); prokhorikhin_aa@almazovcentre.ru (A.A.P.); ahleague@mail.ru (A.O.K.); e.shlyakhto@almazovcentre.ru (E.V.S.); 2 Department of Pharmacological and Biomolecular Sciences, Università degli Studi di Milano, 20133 Milan, Italy; alberico.catapano@unimi.it (A.L.C.); andrea.baragetti@unimi.it (A.B.); 3 IRCCS Multimedica Hospital, Sesto San Giovanni, 20099 Milan, Italy

**Keywords:** multi-omics, risk prediction, cardiovascular disease, precision medicine

## Abstract

Genetics and environmental and lifestyle factors deeply affect cardiovascular diseases, with atherosclerosis as the etiopathological factor (ACVD) and their early recognition can significantly contribute to an efficient prevention and treatment of the disease. Due to the vast number of these factors, only the novel “omic” approaches are surmised. In addition to genomics, which extended the effective therapeutic potential for complex and rarer diseases, the use of “omics” presents a step-forward that can be harnessed for more accurate ACVD prediction and risk assessment in larger populations. The analysis of these data by artificial intelligence (AI)/machine learning (ML) strategies makes is possible to decipher the large amount of data that derives from such techniques, in order to provide an unbiased assessment of pathophysiological correlations and to develop a better understanding of the molecular background of ACVD. The predictive models implementing data from these “omics”, are based on consolidated AI best practices for classical ML and deep learning paradigms that employ methods (e.g., Integrative Network Fusion method, using an AI/ML supervised strategy and cross-validation) to validate the reproducibility of the results. Here, we highlight the proposed integrated approach for the prediction and diagnosis of ACVD with the presentation of the key elements of a joint scientific project of the University of Milan and the Almazov National Medical Research Centre.

## 1. The Need of in Depth Cardiovascular Risk Prevention

The most common forms of cardiovascular disease, presenting with atherosclerosis as the etiopathological factor (ACVD) (e.g., secondary prevention patients complicated by metabolic alterations, severe heterozygous forms of Familial Hypercholesterolemia (FH) or, even more severe, homozygous FH) were untreatable using classical lipid lowering treatments, before the use of genetic information and tremendous the advances in both biotechnological and pharmaceutical research. These advances, that have occurred in recent years, both heralded new therapeutic horizons and contributed further knowledge on the pathophysiological bases of such diseases, making it possible to identify markers at every stage of a molecular or cellular pattern, that can help to cluster patients who require the earliest and most aggressive forms of intervention.

The latter is of immediate interest since, persisting through to today, the multitude of tools currently available in the clinics for risk assessment and the prevention of fatal and non-fatal cardiovascular events [1] are often inaccurate. When addressing the issue of secondary cardiovascular prevention, for example, despite numerous attempts, a tool for a more precise risk assessment in terms of cardiovascular complications has not yet been implemented in clinical practice. Within this group of patients there may be the potential for heterogeneity in the recurrence of cardiovascular events, which represents a particular concern. This can be viewed as a causative factor for subsequent studies focusing on the patient’s position in secondary ACVD prevention.

ACVDs, for all degrees of risk and at every stage, are diseases encompassing a multitude of complex, hardwired biological systems. Thus, in attempts to find a solution for a more accurate risk assessment, numerous factors should be taken into account and blended together, including not only the clinical phenotype of patients, laboratory and instrumental findings, but also the impact of external environmental factors that characterizes the daily life of a person. For example, the classical “metabolic-centric” vision of unhealthy dietary habits in the development of atherosclerosis and the related ACVD risk has been recently implemented in an immune-inflammatory context and is able to induce long-lasting changes in the gene expression and activation of entire molecular systems of the host. In fact, a low quality in dietary habits (e.g., elevated consumption of industrially processed foods in place of fibers and vegetables), which reflects the environmental and socioeconomic status of the subjects [2], induces a long-term somatic leukemogenic expansion of the hematopoietic stem cells, and has been associated with ACVD [3]. An analysis of gut microbiota composition conducted through the use of metagenomic sequencing, recently revealed that the variety of specific bacterial species, associated and implicated with subclinical atherosclerosis [4] and its clinical manifestation [5], predisposes individual metabolic and inflammatory responses to foods [6].

In exome sequencing, epigenomic characterizations are only a part of the larger body of evidence published during recent years proving that multiple novel biomarkers, detectable or measurable only by harnessing forefront high throughput techniques, are necessary for the adoption of a new perspective on ACVD risk assessments. Although on one hand, the richness of information emerging from such pioneering studies promises future effective and personalized tools, on the other hand it presents a challenge of establishing a paradigm for clinicians and translational researchers to improve their perspectives for a broad range of factors during diagnostic and prognostic phases.

The “systems biology” approach, explaining the evolution of ACVD by integrating the nuclear, cellular components, proteins, enzymes generates large data sets that measure numerous analytes, requiring an organized and systematic computational support [7]. Furthermore, undistorted data provide a reliable impact assessment of certain factors, hence making it possible to evaluate the interpretation of each factor and its importance. The inadequate selection of estimated parameters may affect the prognosis of the disease and may lead to an incorrect assessment of the clinical evolution of the atherosclerotic process. It can also result in a mismatch between the individual risk level of the patient and the corresponding medical strategy. In order to construct an unbiased scale for the risk assessment of recurrent ACVD, to using those tools that take into account a great number of external and internal factors will be convenient. Individual risk evaluation data may lead to improved medical care in the context of closer observation of patients belonging to the group with an increased risk of recurrent cardiovascular events. It may also improve the awareness of the patient in terms of their disease, cardiovascular prognosis and may subsequently increase their motivation for lifestyle changes and therapy adherence. Clinical outcomes are more likely to improve if the necessary tools for a risk assessment in a patient population, suitable for secondary disease prevention or at highest risks associated with ACVD.

Finally, it is important to realize that each individual approach does not provide a complete coverage of all the mechanisms that are essential to ACVD progression. Due to the considerably extensive variety and complexity of profiling methods, the choice of the optimal technique(s) in the clinical settings is difficult. It is advisable to refer the obtained data for consequent analysis, taking into consideration the specificity of different “omics” levels. This approach will allow for the confirmation of or for refining test results based on a single data type with an additional review of the information obtained from the same sample set.

## 2. How to Correctly Identify High ACVD Risk Patients? Lessons from Genetics

**Figure 1:**
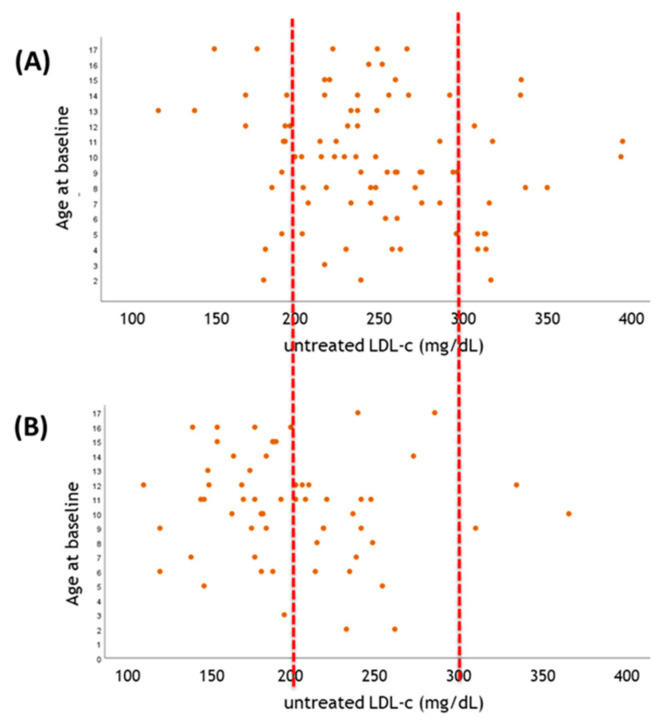
The value of genetic testing in parallel to LDL-C. (**A**) Pediatric FH subjects (age < 18 years-old) carrying LDLR negative FH-mutation (c.1646G > A p.Gly549Asp), displaying median pre-treatment LDL-C level of 249.5 ± 54.0 mg/dL; (**B**) Pediatric FH subjects carrying LDLR defective FH-mutation (c.1775G > A p.Gly592Glu), displaying median pre-treatment LDL-C level of 198.2 ± 50.7 mg/dL. In both panels, graphs correlate the LDL-C before starting statin treatment (*x* axis) vs. the biological age of the probands at basal clinical diagnosis (coinciding with the entry in the LIPIGEN registry following genetic analysis by NGS). Representative dashed red lines help to figure out changes in LDL-C distribution between subjects in both graphs.

The early identification of causal factors and their correction is a crucial part of ACVD risk prediction and prevention. At least 25 dyslipidaemic forms that are associated with either elevated LDL-C, elevated triglycerides or reduced HDL-C have been identified as following the patterns of autosomal dominant or recessive inheritance [8]. Of all of them, FH is the first clear example for which the identification of the genetic etiology contributes to effective prevention. FH is a common genetic disease with an autosomal codominant inheritance pattern, due to the pathogenic mutations in loci encoding for the key factors in cholesterol metabolism (e.g., LDLR, PCSK9 or APOB, APOE and LDLRAP1 genes) [9]. The clinical diagnosis of FH is often driven by clinical algorithms, such as the DLCN criteria, based on the personal and family history of high LDL-C and premature ACVD as well as the detection of premature corneal arcus and tendon xanthomas [9]. In the presence of a probable or definite FH diagnosis, a genetic analysis is recommended, to assess the presence of a causative mutation on candidate genes and to identify a monogenic form, outlining the opportunity for a cascade screening in the family [9,10]. Thus, the identification of causal genetic variants contributing to elevated LDL-C will actually enable an early diagnosis and an effective treatment to reduce the LDL-C burden that characterizes the probands since conception. Besides the already consolidated knowledge on these monogenic diseases, the clinical practice unmasks a significant variability in LDL-C of FH patients harboring the same genetic variants, implying that in a good proportion of patients, the monogenic etiology diagnosis does not completely explain the phenotype of the patients. A clear example of this concept derives from a complete NGS of FH-associated genetic loci over 1532 pediatric FH individuals from the Italian LIPIGEN (“LIpid TransPort disorders Italian GEnetic Network” [11,12]). This experience revealed that FH probands of the same causative variants on LDLR display an elevated variability in the LDL-C levels before starting statin treatment. In addition, a significant number of FH probands of LDLR defective variants (inducing less than 30% of residual LDLR activity, Figure 1A) showed similar LDL-C levels compared to those of the FH probands carrying LDLR negative variants (leaving no more than 5% of the residual activity, Figure 1B).

Even though the importance of genetic factors in the development of ACVD has been proven without reliance on novel fundamental achievements in the study of the human genome, ACVD is viewed as a multifactorial entity [13]. There are monogenic diseases, for which the onset is mainly determined by single gene mutation, as well as polygenic diseases (which include the vast majority of the causes for ACVD), characterized by a combination of variants of several genes and their individual combination. In genetic studies, both monogenic defects and the assessment of polygenic risk scores should be considered. Polygenic risk scores combine the overall influence of several possible genetic variants in the genome and their use for disease prediction [14].

The use of genetic information to stratify monogenic or polygenic causes of FH and the existence of different management care-pathways, is an example of the utility of genetics in precision medicine [15]. As NGS becomes easier to access and as bioinformatics analyses have further developed, this may expand to whole genome sequencing to provide an individual with a more complete picture of their future risk of disease. The achievement and the combination of different genomic techniques can forecast the tissue expression of the key metabolic player (LDLR in this case) for which the phenotype may help to identify genetic relationships between disparate disease. Notwithstanding other models of genomic data have been developed to explore “peripheral genes” in which the up or down-regulation, although not biologically involved in the pathway of the disease, contributes to an increased risk of the diseases, because of perturbations in regulatory gene networks that are co-segregated during inception [7]. The identification of such networks is of particular interest for the identification of the most severe forms of FH or in the identification of subjects that, beyond both LDL-C and a well-characterized monogenic form causal of the disease, harbor an additional genetic contribution of a higher risk of ACVD.

Hence, the possibility to build a “Polygenic Risk Score” that combines both rarer monogenic mutations and more common SNPs causing an LDL-C and ACVD risk score (or both of them separately) represents a valuable process through which to support the therapeutic management of a polygenic etiology in mutation-negative patients with a clinical diagnosis of FH.

In contrast to FH, which represent rare genetic conditions, the association between a specific risk factor and disease onset and progression in large populations does not demonstrate its causal implication for the disease course and prognosis.

**Figure 2:**
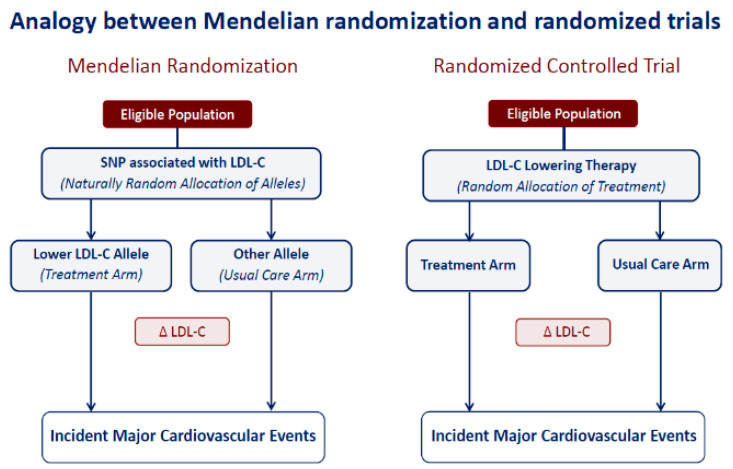
A Mendelian randomization study is analogous to a randomized trial.

The MR approach is a genetic tool that assesses the relation between a genetic variant (or a set of genetic variants with significant probability of linkage disequilibrium) and verifies whether this association significantly predicts the risk of the disease. Through an analogous design to that of randomized clinical trials (Figure 2), the MR thus establishes and assesses the observed relationship between factor influence and a causal relationship [16], demonstrating the ability to assess the impact of genetically determined exposures throughout human life trajectory, excluding the “reverse causality” or the pressures contributed by other risk factors or confounders [17,18].

The example shown here is related to ACVD risk. Numerous genetic variants are associated with lower plasma LDL-C. Each of these variants is allocated randomly at the time of conception in a process referred to as Mendelian randomization. Therefore, inheriting an allele associated with lower LDL-C is analogous to being randomly allocated to LDL-C lowering therapy at birth, while inheriting the other allele is analogous to being randomly allocated to usual care. Because allocation is random, the only difference between the two groups should be their LDL level. As a result, this study design provides a naturally randomized estimate of the benefit of lowering LDL-C early in life analogous to a long-term randomized trial.

MR have shown that long-term exposure to lower LDL-C is associated with a much greater reduction in the risk of cardiovascular events as compared to the same reduction in LDL-C achieved with medications started much later in life as evaluated in randomized trials. This finding implies that the causal effect of LDL-C accumulates over time. Thus, targeting the causes of disease whose effects accumulate over time has the potential to produce much greater reductions in the lifetime risk of ACVD as compared to initiating therapies later in life [16]. Despite its significant relevance to imply causality between a risk factor and the outcome, the interpretation of such genetic techniques needs to be integrated with the clinical experience. A striking example of a distinct relationship between cardiovascular risk factors and outcome was demonstrated in the large UK Biobank registry (438,952 participants; replicated in the Coronary Artery Disease Genome-Wide Replication and Meta-analysis (CARDIOGRAM) plus the Coronary Artery Disease (C4D) genetics consortium (CARDIoGRAMplusC4D)), where both the genetic reduction of systolic blood pressure (SBP) and of LDL-C provided a proportional, linear association with a reduction in the ACVD risk [17]. After calculating two independent scores summing the effect of multiple genetic variants on either SBP or LDL-C, it could be demonstrated that a lifetime exposure to the combination of both lower SBP and lower LDL-C increases the level of the individual ACVD risk.

## 3. Multi-Omics Tools for Cardiovascular Risk Prediction Tools: Transcriptomic and Epigenetic Markers

Multi-omics techniques, that are able to generate large, multidimensional data and to overcome a mono-compartmental approach that provides a unilateral view of an outcome, represent an appealing key strategy to disentangle the highly complex pathophysiology of ACVD [7]. There is a compelling and evident necessity of such techniques since the current approaches to find causality between a marker and ACVD risk, despite providing solid evidence, are affected by important *a priori* limitations. For example, genetic studies and MR approaches shed light on a large number of genes and loci that are associated with, or are supposedly causal for, the risk of the disease. However, the mechanisms by which these genes influence the risk of cardiovascular disease cannot be properly addressed by these approaches, as most of the risk variants associated with CAD or other atherosclerotic ACVD [19,20,21,22,23,24] identified by GWAS and MRs, are often located in noncoding regions of the genome (either intronic or intergenic). In particular cases of such variants, it is clear that they might affect cis or trans regulatory elements that bind transcription factors, enhancers or promoters regulating the expression of specific genes [25]. In response to such limitations, valuable multi-omics connecting genetics to downstream proteomic or metabolomics cellular landscapes might provide further insights on the relation of causality. For example, a GWAS and TWAS on tissue samples from healthy subjects and CAD patients and patients affected by other metabolic diseases enrolled in the multi-ethnic Million Veteran Program study in 2018 investigated the relation between genomic data and lipoprotein levels and lipidomic profiles. In this study, specific variants in genetic regions coding from proteins that could be targeted by forefront biotechnological drugs (PCSK9 and ANGPLT4, for example) were identified and used in a phenome-wide association study (PhWAS) of electronic health record data to identify the lifelong effect of each SNPs on other diseases [26]. This multi-omic approach identified, for example, that the genetic modulation of PCSK9 might have an effect on the transcriptomic profile of the vasculature, representing a valuable example of how integrative omics analyses can develop hypotheses for new potential therapeutic strategies or for drug repurposing. Also, in a different study, an integration of genomics, transcriptomic, nuclear magnetic resonance (NMR) metabolomics and lipidomics from blood samples of subjects in the Dietary, Lifestyle, and Genetic determinants of Obesity and Metabolic syndrome (DILGOM) identified core genetic granulocytes and a mastocytes-lipid network for which the expression correlated with up to 83 metabolites and markers of systemic inflammation [27], contributing further knowledge to new potential areas of investigation.

Additional “multi-omic” studies for CAD, integrated these findings with data from global available tissue transcriptomic, harnessing the analysis of eQTL (genomic loci that explain variation in expression levels of the messenger RNA (mRNA) [28]). Using this tool, different genetic markers have been identified as possible indicators of increased susceptibility to atherosclerosis. For example, BCAR1 has been demonstrated to be causal gene for the faster progression of ultrasound-based Intima-Media Thickness (IMT) in two independent epidemiological studies. In fact, In the the multi-Centre “IMT-Progression as Predictors of Vascular Events” (IMPROVE) cohort and the Italian “Progressione delle Lesioni Intimali Carotidee” (“PLIC”) [29] a hit SNP at the BCAR1-CFDP1-TMEM170A locus emerged via GWAS in both cohorts and increased the BCAR1 eQTL expression in vascular tissues [30]. In a different study evaluating up to 987 public available genomics and transcriptomic datasets, a defined gene network responding to interleukin 1-beta stimulation was found in human smooth muscle cells [31], indicating that such “multi-omics” approaches can provide functional insights into the critical processes of atherosclerotic plaque stability.

High-throughput technologies have been further developed to integrate omics data for the identification of causal genes and molecular mechanisms involved in the development of cardiovascular events. These techniques were applied both in mice, enhancing our understanding about the differential cell function across tissues [32,33,34], and in humans, taking advantage of the large consortia, autopsy evidence and imaging techniques [35,36,37,38].

There is an extensive body of literature linking genetic variations with gene expression and environmental factors that lead to changes in gene expression (“epigenetics”) which contribute to an = understanding of the potential mechanisms of the identified DNA variants in disease manifestation. A clear example is the 9p21 locus, that contains several genes including CDKN2A (encoding cyclin p14, p16), CDKN2B (encoding cyclin p15), MTAP (encoding methylthioadenosine phosphorylase), and the long non-coding RNA enhancer ANRIL. Among these markers, ANRIL in particular showed to be the leading candidate contributing to the relationship between 9p21 regions and CAD, since a transcriptomic analysis of circulating leukocytes demonstrated that the expression of short variants of ANRIL increased by 2.2 fold whereas the expression of the long ANRIL variants decreased by 1.2 fold in healthy subjects homozygous for the risk allele. This finding appeared to be of particular relevance for atherogenesis since the genome-wide expression profiling demonstrated the upregulation of up to 97 genes in carriers of the risk allele; these genes were related the vascular endothelial growth factor for signaling, TNF/MAP kinase pathway signaling in effector T cells, and the interferon response, suggesting that such epigenetic activations promote immune cell trafficking towards inflamed tissues and cellular proliferation [39]. Subsequent in vitro and functional studies supported these predictions, as alleles at the 9p21 locus determining linear transcripts of ANRIL were associated with atherosclerosis while circular transcripts resulted in the protection against atherosclerosis [40]. Furthermore, ANRIL was also demonstrated to be involved in endothelial cell functions, because its expression was downregulated in coronary arteries of CAD patients and promoted the adhesion and trans-migratory potential of monocytes on endothelial cell layers as compared to that which was observed in subjects without CAD [41].

From this perspective, the paradigm of gene-transcript-protein(s) provided by the Human Genome Project must now be transformed into a more “holistic” vision, that includes environmental interactions that affect genomic expression. This change of perspective paves the road towards the understanding of a variety of epigenetics pathways, to unveil how changes in the individual lifelong trajectory of ACVD risk can be either modulated by the environment or can be prevented through the identification of targeted sets of measurable biomarkers.

Aside from non-coding RNAs, other epigenetics modifications have been indicated as potent markers of an enhanced ACVD risk, principally including changes in the DNA histones structural modifications (e.g., methylations/acetylations, consisting of a transfer of a methyl- or acetyl-group to carbon 5 of the cytosine residues [5-methylcytosine (5mC)] in CpG dinucleotides sites). In fact, emerging evidence supports the idea that epigenetic modifications are involved in the initiation and progression of atherosclerosis [42]. Different epigenetic targets have been observed in experimental models of atherosclerosis. For example, the hyper-methylation of the aldehyde dehydrogenase 2 gene (ALDH2) promoter, by downregulating the activity of ALDH, promotes myocardial injuries in rats [43]. Similarly, further epigenetic markers have been underscored in humans. A genome-wide DNA methylation and gene ontology analysis of leukocytes identified four differentially methylated sites in individuals who had a previous MI as compared to the rest of a population without MI; in the same study, a significant correlation between differences in DNA methylation in blood cells and the myocardial expression of Growth Differentiation Factor 15 (GDF-15) (a cytokine involved in regulating apoptosis, cell repair and cell growth) was discovered in MI patients [44].

**Figure 3:**
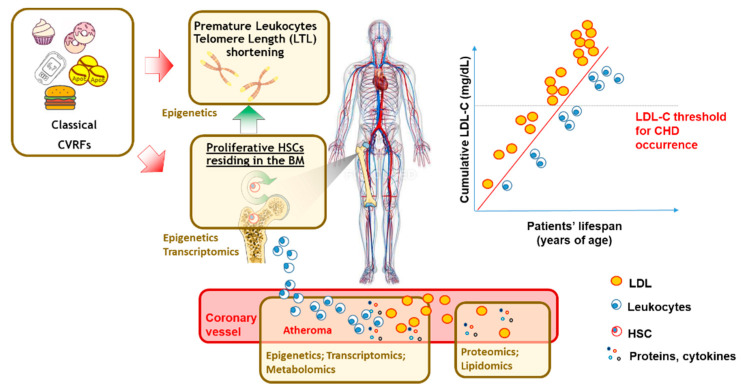
Applicability of “-omics” to prevent the elevated LDL-C burden and the hematopoietic expansion associated with elevated ACVD risk. Green box indicates the tissues, the cells and/or the molecular markers that can be characterized by different -omic approaches. “BM” = Bone Marrow; “CHD” = Coronary Artery Disease; “CVRFs” = Cardiovascular Risk Factors; “HSCs” = Hematopoietic Stem Cells; “LDL” = Low Density Lipoprotein.

The exposure to ACVD risk factors has also been prominently associated with alterations in hematopoiesis [45] and damages in the chromosome ends, leading to supra-physiological DNA telomere length shortening (Figure 3).

Telomeres are DNA-protein complexes that protect chromosome ends from degradation and fusion, thereby regulating the cellular lifespan. In humans, the TL of blood leukocytes (LTL) is inversely associated with ACVD risk factors [46], the onset and progression of several chronic conditions associated with elevated ACVD risk (including diabetes, metabolic syndrome [46]) as well as with risk CHD [47,48] and pre-clinical carotid atherosclerosis [49]. Telomerase, a holoenzyme that adds new telomeric sequences at the end of chromosomes to preserve the telomere length, is very active in high-turnover cells and contributes to the telomere length (TL) maintenance in germ and in progenitor stem cells of leukocytes. Telomere length dynamics thus mirrors the stem cell turnover and hematopoiesis simultaneously [50] and it is therefore plausible that accelerated LTL might reflect an aberrant clonal somatic hematopoietic expansion in atherosclerosis [51]. This lifelong elevated LDL-C burden, causative for elevated ACVD risk since inception and due to somatic mutations in LDLR analyzed by NGS (FH patients), results in an accelerated LTL shortening and reduced blood hematopoietic precursors, even in early life [52]. In addition, transcriptomic profiling of the hematopoietic stem and progenitor cells from bone marrow specimens of FH patients displays an elevated activation of genes involved in pro-inflammatory responses and tissue migrations. All together, these findings suggest possible future translational approaches for the treatment of high ACVD risk patients, such as the FH in this case. Such approaches, by harnessing “-omic”, will have the intent to both efficiently treat classical cardiovascular risk factors, using ongoing therapeutic approaches with more specificity for the patient, and to improve prevention by inviting further research perspectives on novel molecular and cellular markers (Figure 3).

## 4. Multi-Omics Tools for Cardiovascular Risk Prediction Tools: Proteomics

Further large-scale “omics” technologies, covering additional scientific areas other than genomics, allow for the conduction of investigation of susceptibility to faster disease progression and a worse prognosis in the general population. In fact, the positive experience of omics technologies used in the study of ACVD has been demonstrated [7] using genomics [53], but also using transcriptomics [54], on an integrated targeted or non-targeted approach of large proteomic [55], metabolomic [56,57] or lipidomic [58,59] panels (Figure 3). Far beyond the classical analyses of single markers in small-sized cohorts, such approaches have significant implications for ACVD risk prediction leveraging on large multi-Centre consortia where the associations between sets of proteins and the ACVD outcomes that are discovered in a cohort can be validated in others. For example, by using novel technologies, a simultaneous assessment of a large number of biomarkers allowed for the identification of a significant set of up to fifty circulating immune-inflammatory proteins, that were superior, to predict atherosclerotic CV events in the primary prevention setting of the European Prospective Investigation (EPIC)-Norfolk study and to predict the progression of subclinical carotid atherosclerosis in the Italian PLIC study, when considered alongside traditional risk factors [60]. Proteomic sets can therefore represent comprehensive atlases to both improve the understanding of disease pathogenesis and to assist with the identification of patients with a lifelong ACVD risk. In addition, findings from proteomic multi-Centre consortia speculate that a pathway analysis of the proteomic signature may also allow for the guidance of the most appropriate medication to use in specific patient categories [7,55], a concept that has been adequately explored by the experience of the CANTOS study, where, predominantly CRP responders demonstrated the CV benefit of anti-interleukin 1 beta-antibody administration (Canakinumab) [61] and in the LoDoCo2 Study, where colchicine treatment promoted the potent reduction of the innate immune-inflammatory response [62].

## 5. Cardiovascular Risk Prediction Tools: The Opportunity to Use Artificial Intelligence/Machine Learning Approaches

Once -omics are obtained from experimental platforms, the large amount of data requires a predefined and coordinated analysis that should rely on the specificity of applied approaches. A targeted approach allows for the creation of distinct metabolite profiles with defined chemical and biochemical characteristics. However, the principal disadvantage of this method is the limited availability of detectable markers, which may not include other more predictive markers. This can only be solved using an interdisciplinary approach, including the collaboration of clinicians, biochemists and bioinformaticians, with the development of a dedicated, convenient and clear software in the real-world clinical practice. Processing a large amount of data with traditional software can be overwhelming due to both the volume and the variety of data types. High-tech big data analysis tools such as AI software and ML can be viewed as key in the determination of a significant correlation between variables. The use of AI and ML systems can act as a valuable resource for medical professionals, allowing them to study complex and nonlinear hierarchical models in the interpretation of phenotype-genotype relationships, thereby providing a more complete understanding of the course of the pathophysiological processes. Such a set of tools can serve as a “bridge” to new biomedical discoveries and advances in personalized medicine. Combining data from a multi-omics approach with a genetic analysis in ACVD research [63,64,65] can thus provide a more precise risk assessment compared to the use of standard clinical risk factors, promoting the way to disease learning—from genotype to phenotype. Each platform provides measurements of various factors influencing the course of the disease, thereby providing an additional gusset to improve the approach to the prediction of ACVD and subsequent disease prognosis.

However, several critical challenges are to be considered in the management of such an approach. Firstly, non-targeted identification is used to simultaneously measure a large number of potential markers in biological samples. It should, however, be noted that many activity “signals” may not derive from the metabolite, but instead an analysis interference. The incremental implementation of a non-targeted approach followed by the usage of a more precise and targeted analysis can provide a preferable biomarker identification strategy. The combined use of current diagnostic and predictive tools can lead to an understanding of how the gene expression profile affects phenotype and protein expression, including post-translational modifications, as well as the way in which it influences final metabolite profiles. Causal relationships derived from the data set analysis can provide researchers with an opportunity to gain new insights into the mechanisms underlying ACVD pathophysiology. It can present more precise and reliable information about the course of the disease, which is key for a more accurate and effective strategy in individual patient management.

Secondly, the variable selection method presents an issue that might not be completely addressed by the deep learning algorithms, in the quest to categorize representative multi-dimensional data integration studies [66]. Therefore, integrative analyses performed in the supervised, semi-supervised and unsupervised manner, within both parallel and hierarchical integration studies are warranted to test the efficiency of the final AI/ML tool. Finally, the disease traits in complex disease, including ACVD are generally heterogeneous with outlying observations and heavy tailed distributions [67], posing challenges to the reproducibility of the findings. All these criticisms might affect, in different ways, the use of multi-omics integration for ACVD risk prediction in the population or the identification of the rarest forms of dyslipidemia. Rather, multiple predictive algorithms and omics markers might be less appropriate for the estimation of the ACVD risk on a large, population-based scale because of the co-presence of different factors related to the host or to its environmental exposure. Conversely, in rarer forms, fewer signals from the multi-omics integration might appear less likely to be affected by one or few factors that are genetically determined, and that predominate the phenotype.

## 6. Cardiovascular Risk Prediction Tools: A Joint Research Project

**Figure 4:**
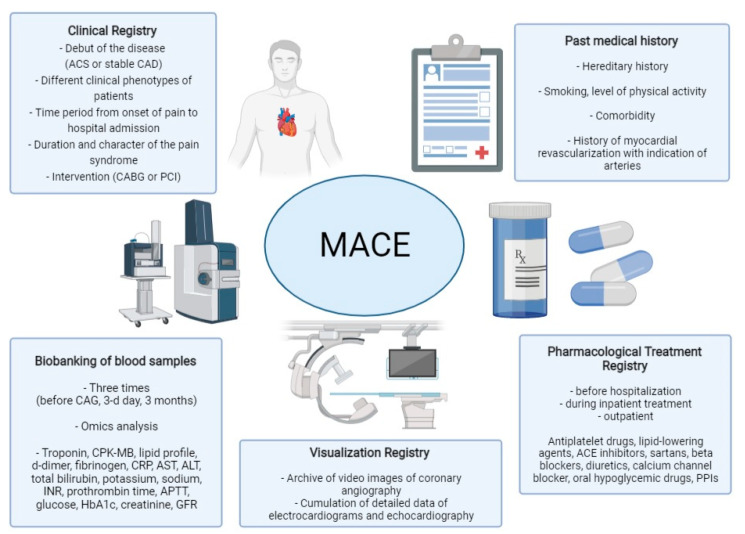
Characterization of the key parameters collected within the Registry. Design of the joint research project between University of Milan and the Almazov National Medical Research Centre. “ACS” = Acute Coronary Syndrome; “APTT” = activated partial thromboplastin time; “AST” = aspartate transaminase; “ALT” = alanine aminotransferase; “CABG” = coronary artery bypass graft; “CAD” = Coronary Artery Disease; “CPK-MB” = Creatine Phosphokinase-MB; “CAG” = Coronary Angiogram; “CRP” = C-Reactive Protein; “GFR” = Glomerular Filtration Rate; “HB1Ac” = Glycated hemoglobin; “INR” = International Normalized Ratio; “MACE” = Major Cardiovascular Event; “PCI” = Percutaneous Coronary Intervention; “PPIs” = Proton-pump inhibitors.

In accordance with an agreement on international cooperation between the University of Milan and the Almazov National Medical Research Centre, a joint research project is underway to use up-to-date methods in the molecular assessment of potential biomarkers for recurrent cardiovascular event predictions in patients with ACS in real-world clinical practice (Figure 4).

The study will include men and women over 18 years old with an established diagnosis of ACS. Prior to their inclusion, all participants will sign an informed consent form. The study will not include pregnant women, patients with known oncological or mental illnesses and patients with end-stage disease. Given the fact that ACS is a heterogeneous group of diseases, patients are divided into three groups: patients with unstable angina pectoris, patients with STEMI and patients with a non-STEMI diagnosis. In each group of patients, information on the onset of the disease will be collected and the disease vintage over time will be calculated. Upon enrolment, if a patient is likely to belong to a group with a long-term history of coronary artery disease, information will be collected regarding the form of CAD and previous myocardial revascularization with a specific emphasis on the affected artery. Personal anamnesis data will also be collected (e.g., nutritional habits, alcohol intake, physical activity, smoking, family history, concomitant diseases and medications, etc.). Venous blood sampling is performed in several steps. The first blood sampling point is performed initially within the first hour prior to CAG. The second sampling point is performed on the third day after inpatient treatment. The third sampling point is three months after the discharge from the hospital. The heart rhythm, conduction abnormalities, Q wave and ST segment morphology are assessed using ECG. Echocardiography helps to assess all routinely measured parameters with an emphasis on the altered local myocardial contractility. According to a CAG data analysis, the type of coronary circulation and the levels of coronary artery lesions are taken into account. Information concerning a patients’ drug therapy prior to hospitalization, during inpatient treatment and on an outpatient basis is also recorded.

This project will include a complete genetic analysis of the dominant variants associated with hypercholesterolemia with an increased ACVD risk. In addition, a complete set of proteomic, metabolomics and lipidomic markers will be also quantitated in samples of circulating plasma. This data may be beneficial as a tool for predicting the recurrence of cardiovascular events. The archive of patient data will be implemented (i.e., complaints, history of the disease, physical examination findings, laboratory parameters, CAG videos) and detailed electrocardiography and the echocardiography results will be collected with the aim of identifying various phenotypic patient profiles and to concomitantly interpret the data obtained using omics technologies. The impact of lipid-lowering therapy on the biomarker levels will also be assessed, taking into account the drug- and especially statin-naivety of the patients.

Collectively, these data will help to improve the efficiency of therapy in patients who have suffered from ACS and to identify a group of patients with a higher risk of recurrence of cardiovascular events. Presenting a new perspective, this strategy will provide progress in the development of a personalized secondary prevention approach.

Beyond the majority of single-time cross-sectional approaches in this field, the longitudinal design of this investigation will highlight the dynamics of expression data and may uncover other additional factors influencing the time course of the disease.

Moreover, regarding the subjects in secondary prevention, this time dependent evaluation will underscore changes that are directly or indirectly initiated by acute cardiovascular events. A prospective approach recording expression profiles at different time cut-offs will provide a more accurate and comprehensive analysis in the context of the cardiovascular disease prolongation.

The predictive in silico models for data analysis of the elevated amount of -omic data will be based on consolidated AI best practices for classical ML and deep learning paradigms to avoid the previously cited criticisms (variable selection method [66] and furthermore, cross-validation can warrant reproducibility [67]). The model will employ methods (e.g., cross-validation) to validate the reproducibility of the results. With this regard, the data sample will be split into training and test sets (usually 80% and 20% respectively). In regard to the training set, we will set up the algorithm and build a powerset of the feature set. Each element of the powerset will be used for training a logistic regression model (fitting the parameters). Then, we will calculate the area under the Receiver Operating Curve (ROC) using the trained regression model and the vector of binary variables. We repeat this procedure for each element in the powerset. The model will use a subset of factors and the vector of their coefficients for a calculation of the test sample. The features from the different omics layers will be integrated in an ML model based on the Integrative Network Fusion method. Both AI/ML supervised and unsupervised strategies will be developed to derive a classification algorithm from the derivation cohort, which will be applied to subsequent validation cohorts to construct an intervention model to identify the greatest number of discriminant biomarkers for response prediction. The algorithm will be refined using an ML-based iteration with additional datasets collected within the project.

A multitude of publicly available clinical datasets and a tissue expression atlas from diverse experimental models have been produced in the recent years which represent an almost fulfilled scientific request. The next objective is the leveraging of informatics and the understanding of systems biology [7] to both estimate the individual risk among large communities and to identify the rarer forms of highest and premature ACVD risk. This appears a feasible aim, that can be achieved by blending multi-omics, genetic approaches and clinical features. At the same time, however, this comprehensive approach should take into account the clinical perspective of and sensitivity to the disease. This joint scientific project actually responds to this request, by connecting translational scientists, who are involved in a well-established Italian network for genetic dyslipidemias, and different clinical entities, lipidologists and cardiologists. By establishing this multi-omic approach, the research joint group aims to export and exchange, on a global basis, this research, providing a paradigm for a new and more accurate level of in vivo staging of the atherosclerotic process. In addition, to provide further translational knowledge in atherosclerosis, this scientific endeavor will act as a proof-of-concept to surmise future clinical tools of personalized medicine to be applied at any stage of ACVD risk.

## Data Availability

The manuscript does not involve data generated by the research joint venture between University of Milan and Almazove Centre.

## Abbreviations

ACS	Acute Coronary Syndrome
ACVD	Atherosclerotic Cardiovascular Disease
AI	Artificial Intelligence
ANGPLT4	Angiopoietin-Like 4
APOB	Apolipoprotein B
APOE	Apolipoprotein E
CAD	Coronary Artery Disease
CAG	Coronary Angiography
CHD	Coronary Heart Disease
CRP	C-Reactive Protein
DLCN	Dutch Lipid Clinic Network
eQTL	Expression quantitative trait loci
ECG	Electrocardiogram
FH	Familial Hypercholesterolemia
GWAS	Genome-Wide Association Studies
HDL-C	High-Density Lipoprotein cholesterol content
LDLR	Low-Density Lipoprotein Receptor
LDL-C	Low-Density Lipoprotein cholesterol content
LDLRAP1	Low Density Lipoprotein Receptor Adaptor Protein 1
MI	Myocardial Infarction
ML	Machine Learning
MR	Mendelian Randomization
NGS	Next-Generation Sequencing
PCSK9	Proprotein Convertase Subtilisin/Kexin type 9
PhWAS	Phenome-wide association study
SNPs	Single Nucleotides Polymorphisms
STEMI	ST-elevation myocardial infarction (STEMI)
TWAS	Transcriptome Wide Association Study
